# A phase 1b study of zilovertamab in combination with paclitaxel for locally advanced/unresectable or metastatic HER2-negative breast cancer

**DOI:** 10.1186/s13058-024-01782-0

**Published:** 2024-02-26

**Authors:** Rebecca A. Shatsky, Hemali Batra-Sharma, Teresa Helsten, Richard B. Schwab, Emily I. Pittman, Minya Pu, Elizabeth Weihe, Emanuela M. Ghia, Laura Z. Rassenti, Alfredo Molinolo, Betty Cabrera, James B. Breitmeyer, George F. Widhopf, Karen Messer, Catriona Jamieson, Thomas J. Kipps, Barbara A. Parker

**Affiliations:** 1 Moores Cancer Center, University of California San Diego, 3855 Health Sciences Drive Mail Code 0987, La Jolla, San Diego, CA 92093 USA; 2 Department of Medicine, University of California San Diego, La Jolla, San Diego, CA USA; 3 Herbert Wertheim School of Public Health, University of California San Diego, La Jolla, San Diego, CA USA; 4 Department of Radiology, University of California San Diego, La Jolla, San Diego, CA USA; 5 Center for Novel Therapeutics, University of California San Diego, La Jolla, San Diego, CA USA; 6 University of California San Diego California Institute for Regenerative Medicine Alpha Clinic, La Jolla, San Diego, CA USA; 7 Oncternal Therapeutics, Inc., San Diego, CA USA; 8 Sanford Stem Cell Institute, University of California San Diego, La Jolla, San Diego, CA USA

**Keywords:** ROR1, Zilovertamab, Paclitaxel, Metastatic breast cancer

## Abstract

**Background:**

Zilovertamab is a humanized monoclonal antibody targeting ROR1, an onco-embryonic antigen expressed by malignant cells of a variety of solid tumors, including breast cancer. A prior phase 1 study showed that zilovertamab was well tolerated and effective in inhibiting ROR1-signaling, which leads to activation of *ERK1/2*, *NF-κB*, and *NRF2* target genes. This phase 1b study evaluated the safety and tolerability of zilovertamab with paclitaxel in patients with advanced breast cancer.

**Patients and methods:**

Eligible patients had locally advanced, unresectable, or metastatic HER2^−^ breast cancer with Eastern Cooperative Group performance status of 0–2 and without prior taxane therapy in the advanced setting. Study treatment included 600 mg of zilovertamab administered intravenously (IV) on Days 1 and 15 of Cycle 1 and then Day 1 of each 28-day cycle along with paclitaxel weekly at 80 mg/m^2^ IV.

**Results:**

Study patients had received a median of 4 prior therapies (endocrine therapy + chemotherapy) for locally advanced, unresectable, or metastatic disease. No patient discontinued therapy due to toxicity ascribed to zilovertamab. Adverse events were consistent with the known safety profile of paclitaxel. Of 16 patients, 6 (38%) had a partial response, and 6/16 (38%) patients had stable disease as best tumor response.

**Conclusion:**

The combination of zilovertamab and paclitaxel was safe and well tolerated in heavily pre-treated advanced breast cancer patients. Further evaluation of ROR1 targeting in breast cancer patients with zilovertamab is warranted.

*Trial Registration*: NCT02776917. Registered on ClinicalTrials.gov on 05/17/2016.

## Background

ROR1 (receptor tyrosine kinase-like orphan receptor 1) is a highly conserved onco-embryonic surface protein that is expressed on the neoplastic cells of many malignancies, including breast cancer [1–5]. Because ROR1 is not expressed on most normal postnatal tissues, it is a potential target for anti-cancer therapy [5–7]. Expression of ROR1 on breast cancer cells enhances activation of cyclic adenosine monophosphate (cAMP), phosphatidylinositol-3-kinase (PI3K)/protein kinase B family (AKT), and non-canonical wingless-type integration site family (Wnt) signaling pathways and has been associated with more aggressive disease and an enhanced metastatic trajectory [8–10]. High ROR1 protein expression by immunohistochemical staining was reported in 40% of breast tumor specimens including 55% of lobular carcinomas and 29% of ductal carcinomas [2]. Other studies found variable results with different anti-ROR1 antibodies [1, 8]. One study found high ROR1 staining in 56% of triple-negative breast cancer samples and low ROR1 staining in 12% of ER^+^PR^+^ samples and no staining in 12 HER2^+^ samples [1]. Expression of *ROR1* transcripts generally correlates with the expression of ROR1 protein [11]. High *ROR1* gene expression in the I-SPY2 transcriptomic dataset was associated with worse event-free survival in hormone receptor (HR)^+^/HER2^−^ patients with high residual cancer burden after neoadjuvant treatment [12].

Our group developed zilovertamab (previously UC-961 or cirmtuzumab), which is a fully humanized monoclonal antibody (mAb) with high affinity and specificity for an extracellular epitope of ROR1 [13]. A phase 1 trial of zilovertamab in patients with chronic lymphocytic leukemia (CLL) found this mAb was well tolerated and effective in inhibiting ROR1-signaling and cancer-stemness gene expression in leukemia cells of treated patients [13]. Preclinical studies in immunodeficient mice bearing breast cancer patient-derived xenografts (PDXs) showed that zilovertamab repressed expression of genes associated with breast cancer stemness, impaired metastases, and inhibited re-engraftment in immunodeficient mice. Additionally, zilovertamab in combination with paclitaxel had additive, if not synergistic, anti-tumor activity in immunodeficient mice engrafted with a breast cancer PDX [14]. Therefore, this phase 1b clinical trial was conducted to evaluate the safety of zilovertamab in combination with paclitaxel in patients with locally advanced, unresectable, or metastatic breast cancer.

## Methods

### Phase 1b study objectives

This was a single center, open-label, phase 1b study evaluating the safety of zilovertamab when used in combination with paclitaxel for treatment of patients with locally advanced, unresectable, or metastatic HER2^−^ breast cancer. The study protocol was approved by the Human Research Protections Program (HRPP) at the University of California, San Diego (IRB #160178, NCT02776917). We obtained written informed consent from each patient prior to study enrollment.

The primary objective was to determine the safety of zilovertamab and weekly paclitaxel in patients with advanced breast cancer, evaluating for dose limiting toxicities (DLTs) of the combination in the first 28-day cycle. Secondary objectives were to assess overall safety, pharmacokinetics, and clinical activity. An exploratory objective was to compare PET/CT to standard cross-sectional imaging in a subset of patients.

### Patient selection

Patients aged 18 years or older with Eastern Cooperative Group (ECOG) performance status 0–2 and adequate organ function were eligible if they had biopsy-confirmed, locally advanced, unresectable, or metastatic HER2^-^ breast cancer with no maximum number of prior lines of therapy. Patients were required to have measurable disease according to Response Evaluation Criteria for Solid Tumors (RECIST) version 1.1 [15]. Patients were excluded if they had prior taxane therapy in the advanced, unresectable, or metastatic setting or known resistance to taxane therapy, defined as refractory to paclitaxel in the neoadjuvant setting and/or developed metastatic breast cancer within 6 months of neoadjuvant or adjuvant taxane chemotherapy. Patients were excluded if they had a concurrent, uncontrolled serious illness, another primary cancer, or had uncontrolled brain metastases or leptomeningeal disease. We excluded patients with preexisting neuropathy of greater than grade 1.

### Treatment plan and assessment

Eligible patients received zilovertamab at a fixed dose of 600 mg IV on Day 1 and Day 15 for the first cycle and then Day 1 of each subsequent 28-day cycle. Paclitaxel was given following zilovertamab at a dose of 80 mg/m^2^ IV weekly. Treatment continued until disease progression or until patients experienced unacceptable treatment-related toxicity. If toxicity was deemed to be related to paclitaxel, patients were allowed to continue single agent zilovertamab. Toxicity and efficacy assessments were performed according to Common Terminology Criteria for Adverse Events (CTCAE) version 4.03 and RECIST version 1.1, respectively [15, 16]. Response was assessed every 8 weeks by cross-sectional imaging with computed tomography (CT) or magnetic resonance imaging (MRI). 18F-fluorodeoxyglucose (18F-FDG) positron emission tomography/computed tomography (PET/CT) scans were obtained on some patients and correlated with cross-sectional imaging.

### ROR1 expression and pharmacokinetics

Available baseline formalin-fixed, paraffin-embedded breast cancer samples were assessed for ROR1 expression via immunohistochemical (IHC) staining, as reported [14]. Optional tissue or malignant fluid was obtained at baseline, between Cycle 3 Day 1 and Day 15, at the time of progression from consenting patients, and when malignant pleural or ascitic fluid was drained for palliation.

Peripheral blood samples for pharmacokinetic analysis were obtained on Days 1 and 15 of Cycle 1, prior to zilovertamab infusion, at 30 min post-completion of zilovertamab infusion, and then immediately after infusion of paclitaxel. On Days 8 and 22 of Cycle 1, blood was obtained prior to the infusion of paclitaxel and during all subsequent cycles, blood was obtained for pharmacokinetic analysis only prior to the infusion of zilovertamab. We determined zilovertamab levels as reported using an ELISA to determine the plasma concentrations of human IgG that was able to bind immobilized ROR1 [13].

### Statistical analysis

DLTs were defined as clinically significant adverse events considered by the investigator to be possibly, probably, or definitely related to zilovertamab or the combination of zilovertamab with paclitaxel within 28 days of investigational treatment initiation. DLTs included Grade 4 hematologic toxicity lasting more than 7 days and non-hematologic toxicity of grade 3 or higher.

Patients were enrolled in cohorts of five, and each cohort was assessed for DLTs prior to enrollment of the next cohort. If two or more patients in the first cohort experienced DLT attributed to zilovertamab at full dose, then the next cohort would be enrolled at a 50% dose reduction (300 mg flat dose). If two or more patients in the second cohort experienced DLT attributed to zilovertamab at a 50% dose reduction, then the study would be stopped. If fewer than two patients in the first cohort experienced DLT attributed to zilovertamab at full dose, but two or more in the second cohort experienced DLT at full dose, then the third cohort would be treated with 50% dose-reduced zilovertamab. For adverse events other than DLTs attributed to study treatment, dose and schedule changes were specified in the protocol. The protocol also specified that for Grade > 2 rash, allergy, or infusion reaction, nab-paclitaxel may be substituted at investigator discretion.

The intent-to-treat population includes all patients who started at least one dose of zilovertamab. Descriptive statistics are used to characterize demographics, safety, toxicities, and anti-tumor activity. Best tumor responses are shown in a waterfall plot, and a swimmer plot is used to show tumor responses for each patient while on study treatment. Confidence intervals of the median progression-free survival (PFS) time are estimated based on Kaplan–Meier estimates, with PFS defined as weeks from the first day of study treatment to first disease progression or death. The duration of partial response (PR) was defined as the time from the first PR assessment to the time of recurrence, progression, or death.

## Results

### Patient demographics

We enrolled 16 patients between August 2018 and May 2021 (Table 1). One patient (BROR-01) discontinued study treatment prior to completion of the 4-week DLT assessment period due to symptomatic bone metastases present at entry that required surgical fixation. The patient was replaced as per protocol to have 15 patients who completed treatment for 8 weeks. All patients enrolled were female with age range of 30–72 and median age of 51.5 years. Race/ethnicity categories included 11/16 (69%) White, 4/16 (25%) self-reported Hispanic, 3/16 (19%) of more than one race, and 2/16 (12%) Asian. All patients had ECOG performance status of 0 or 1. Thirteen patients had visceral metastatic disease at study enrollment, defined as involving soft tissue organs including the lung, pleura, liver, ovary, peritoneum, non-regional lymph nodes, and distant soft tissue (e.g., retroperitoneal mass). Ten patients had previously received CDK 4/6 endocrine combination therapy, and 11 patients had received prior chemotherapy including up to 3 different regimens. At study entry, 10/16 (63%) had ER^+^ and/or PR^+^/HER2^−^ locally advanced or metastatic disease and 6/16 (38%) had ER^−^PR^−^/HER2^−^ disease.

**Table 1:** Patient demographics

BROR#	Age at enroll	Stage at DX	ER at DX	PR at DX	ER at enroll	PR at enroll	Time from DX to 1st met (months)	Disease sites at enrollment	Prior therapies for mets
01*	54	T3N3M0	99% 3+	40% 3+	99% 3+	99% 3+	23	Bone, liver	3-Endo, 3-Chemo
02*	59	T2N2M0	Positive	Positive	Negative	Negative	152	Bone, chest wall, non-regional lymph nodes	1-Endo, 0-Chemo
03	42	T2N1M0	80% 2+	10% 2+	Negative	Negative	34	Bone, chest wall	3-Endo, 3-Chemo
05	41	T2N0M0	Negative	Negative	Negative	Negative	32	Chest wall, lung	1-Endo, 3-Chemo
07*	42	T4N1M0	Positive	Positive	5% 1+	40% 1+	56	Bone, liver, lung	4-Endo, 3-Chemo
08*	59	T1N1M0	50% 2+	50% 2+	25% 2+	40% 2+	77	Bone, lung, retroperitoneal mass	1-Endo, 2-Chemo
09	30	T3N1M0	0%	0%	Negative	Negative	22	Lung	0-Endo, 0-Chemo
11	72	T2N1MO	30% 1+	30% 1+	5% 2+	85% 1+	27	Chest wall	0-Endo, 0-Chemo
12	54	T2NOMO	6%	0%	Negative	Negative	17	Lung	0-Endo, 0-Chemo
13*	69	T1N0M0	Positive	Positive	95% 3+	20% 3+	108	Liver, non-regional lymph nodes	3-Endo, 2-Chemo
14*	46	T1N2M0	99% 3+	43% 2+	60% 3+	5% 1+	51	Bone, chest wall	2-Endo, 2-Chemo
15*	64	TXN1M1	95% 3+	50% 2+	99% 3+	99% 3+	Less than 1	Bone, liver	3-Endo, 1-Chemo
16*	63	T3N2M0	99%	90%	85% 3+	40% 1+	72	Peritoneum, pleura	2-Endo, 3-Chemo
17*	49	T4N3M0	60%	40%	80% 2+	Negative	37	Bone, liver, non-regional lymph nodes, ovary, pleura	2-Endo, 2-Chemo
21	49	T1N0M0	Negative	Negative	Negative	Negative	29	Chest wall, lung	0-Endo, 0-Chemo
22*	48	T3N1M0	95% 3+	95% 3+	100%	< 1%	47	Chest wall, Lung	1-Endo, 3-Chemo

If the pathology report stated “Negative” or “Positive” to describe the immunohistochemical expression of ER and PR, this is noted instead of quantified expression

*Chemo* chemotherapy, *DX* diagnosis, *Endo* endocrine therapy, *Enroll* enrollment, *ER* estrogen receptor, *Mets* metastases, *PR* progesterone receptor

*Indicates prior CDK4/6 inhibitor therapy

### Safety

All 16 patients were evaluable for safety and tolerability of the combination of zilovertamab and paclitaxel, and fifteen patients completed the 28-day DLT assessment. We observed no DLTs. The most common adverse events (AEs) attributed to study therapy were fatigue (*n* = 13/16 [81% of subjects]), nausea (*n* = 11/16 [69% of subjects]), and peripheral sensory neuropathy (*n* = 8/16 [50% of subjects]) (Table 2). The most common reported AE of any grade attributed to zilovertamab was nausea (*n* = 2/16 [13% of subjects]).

**Table 2:** Most common adverse events (AEs) attributed to study therapy

	AE name	Number of events	Number of subjects	% of subjects	Grade 1	Grade *2*	Grade 3	Grade 4
1	Fatigue	14	13	81	13	1	0	0
2	Nausea	12	11	69	11	0	0	0
3	Peripheral sensory neuropathy	8	8	50	6	2	0	0
4	Neutrophil count decrease	7	6	38	0	1	5	1
5	Peripheral motor neuropathy	7	6	38	7	0	0	0
*6*	Constipation	6	6	38	6	0	0	0
7	Alopecia	6	6	38	5	1	0	0
8	Dyspnea	5	3	19	5	0	0	0
*9*	Diarrhea	4	4	25	4	0	0	0
10	Flu like symptoms	4	3	19	3	0	1	0

Six patients experienced Grade 3 or 4 treatment-related adverse events (TRAEs). One patient (BROR-01) had two episodes of grade 3 neutropenia considered possibly related to zilovertamab. The remainder of the AEs attributed to zilovertamab were grade 1 or 2. All other grade 3 or 4 TRAEs were documented as definitely related to paclitaxel; these included grades 3 or 4 neutrophil count decrease in 4 patients, one patient with grade 3 anemia, and one patient with grade 3 flu-like symptoms. No dose adjustments or schedule delays of zilovertamab were required due to adverse events.

Except for one patient (BROR-01) who discontinued study treatment early due to clinical progression as explained above, the remaining 15 patients continued zilovertamab until disease progression. Three of the 15 patients continued zilovertamab alone after discontinuing paclitaxel early due to paclitaxel-induced rash, peripheral neuropathy, or patient preference; the remainder continued combination therapy until disease progression. No patient received nab-paclitaxel.

### Pharmacokinetics

**Fig. 1:**
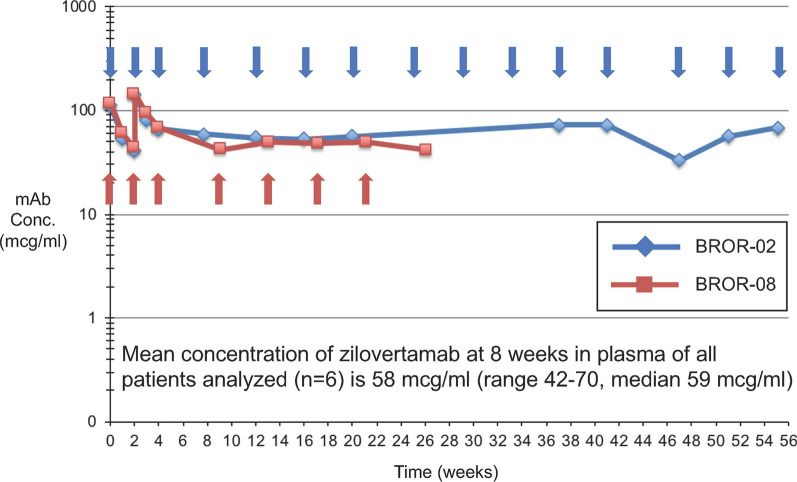
Zilovertamab concentration in plasma of representative patients. Zilovertamab concentration (mcg/mL) is indicated on the *y* axis, and time (weeks) is indicated on the *x* axis. Arrows indicate days of infusion of zilovertamab

**Fig. 2:**
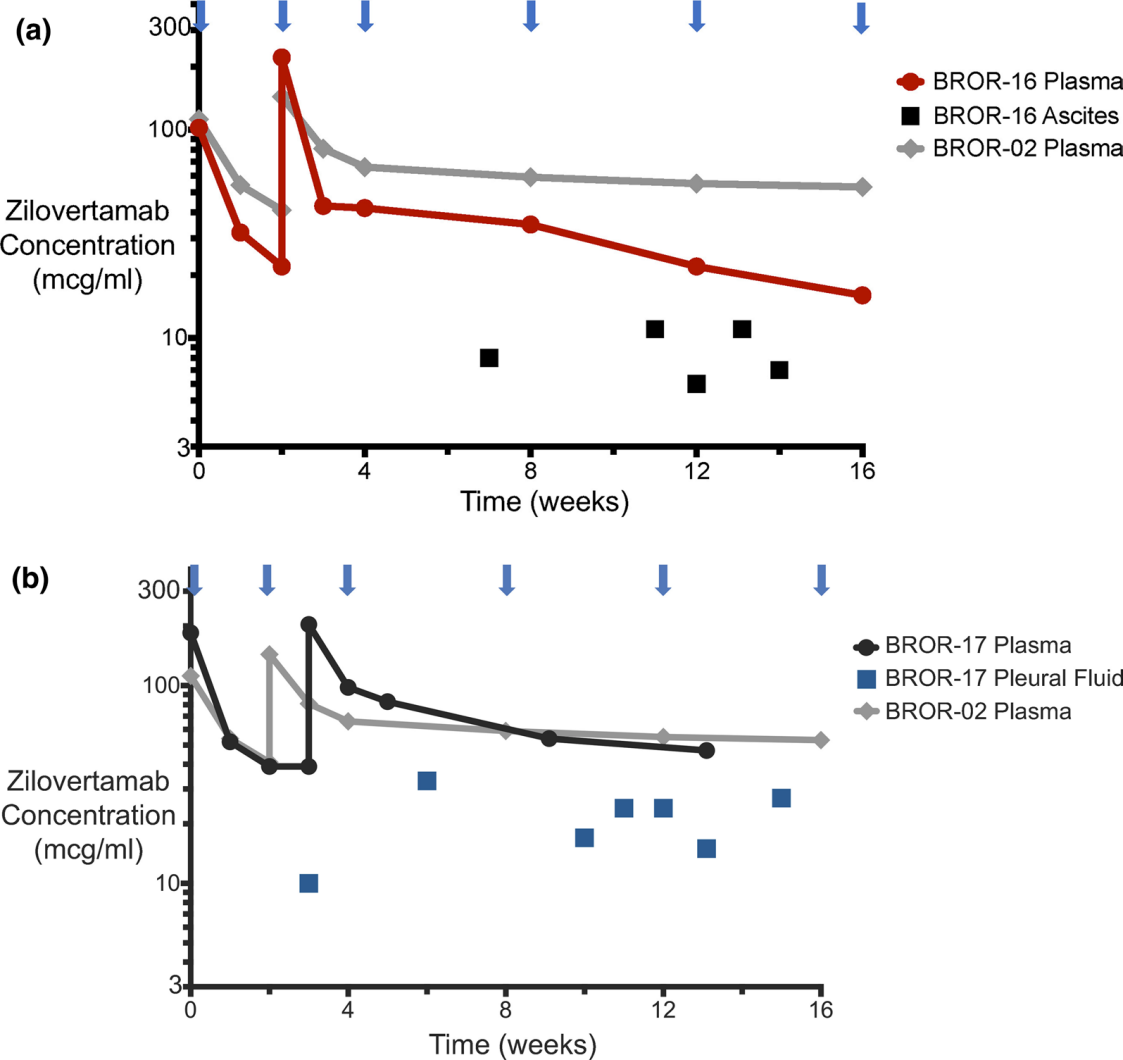
**A** Pharmacokinetics in ascites versus plasma in BROR-16. **B** Pharmacokinetics in pleural fluid versus plasma in BROR-17. Zilovertamab concentration (mcg/mL) is indicated on the *y* axis, and time (weeks) is indicated on the *x* axis. Arrows indicate days of infusion of zilovertamab. BROR-02 plasma levels are included as representative of the mean concentration of zilovertamab of 58 mcg/ml among 6 patients analyzed at 8 weeks (see Fig. 1)

Pharmacokinetic analysis of blood from 6 patients revealed a median plasma zilovertamab level of 58 µg/mL prior to the 2nd or 3rd cycle infusions of zilovertamab (Fig. 1). The estimated half-life of zilovertamab was at least 28 days (Fig. 1). Analysis of pleural or ascitic fluid in 2 patients showed zilovertamab levels of approximately 30% of those in plasma (Fig. 2A, B). Patient BROR-2, BROR-8, and BROR-17 had similar plasma levels of zilovertamab. BROR-16 had lower levels of plasma zilovertamab.

### Efficacy

**Fig. 3:**
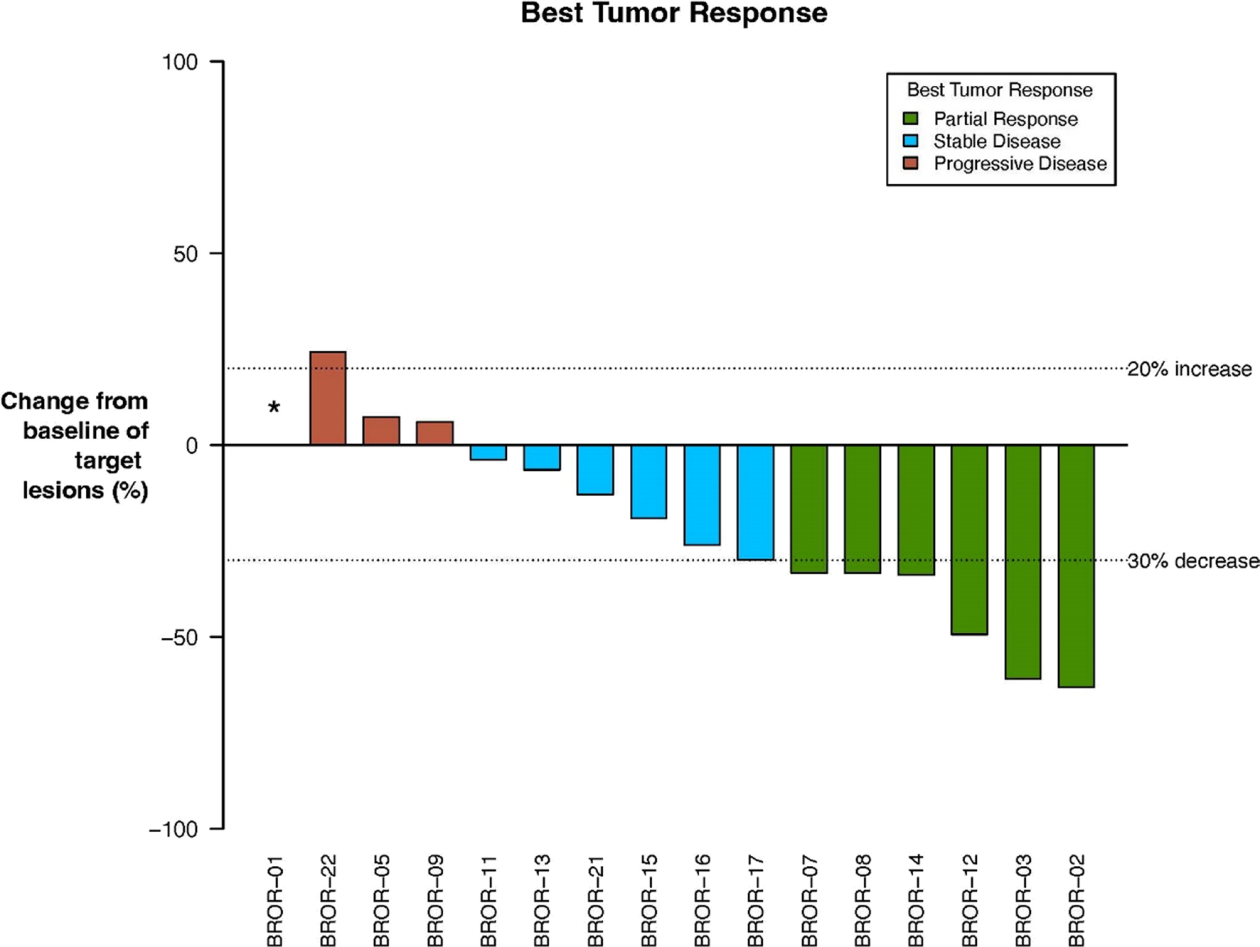
Best tumor response. Best tumor responses are shown using colored bars in this waterfall plot. The y-axis reflects percentage change in maximal tumor size compared to baseline in target lesions, and bar colors indicate overall best response. *BROR-01 had clinical progression 3 weeks after treatment initiation requiring study discontinuation before the first imaging assessment

Efficacy evaluation of the 16 patients receiving the combination of zilovertamab and paclitaxel in the intent-to-treat population revealed 6/16 (38%) patients with partial response, 6/16 (38%) with stable disease, and 4/16 (25%) with progressive disease as best response (Fig. 3).

**Fig. 4:**
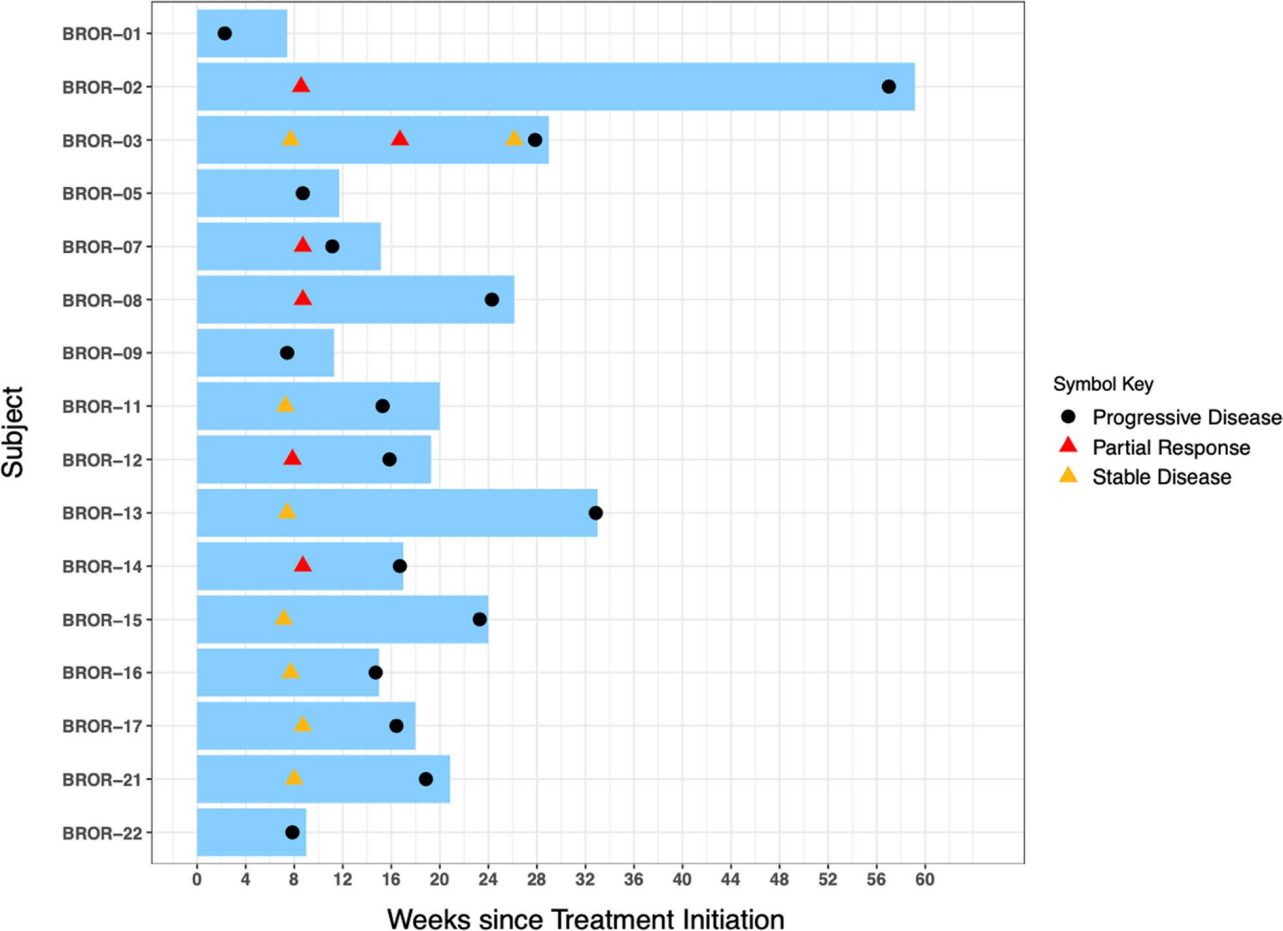
Duration of treatment for intention-to-treat population

No patient had a complete response. Median PFS was 16.1 weeks (95% CI 11.1–23.3 weeks). Median number of cycles of treatment received was 4 (range 1–14). Median number of doses of zilovertamab received was 5 (range 1–15), and median number of doses of paclitaxel received was 15 (range 2–31). One patient (BROR-02) had a PR of 48 weeks (Fig. 4), with duration of zilovertamab and paclitaxel therapy for 23 weeks, followed by zilovertamab alone for 32 weeks (paclitaxel discontinued due to peripheral neuropathy).

### Correlative studies

**Fig. 5:**
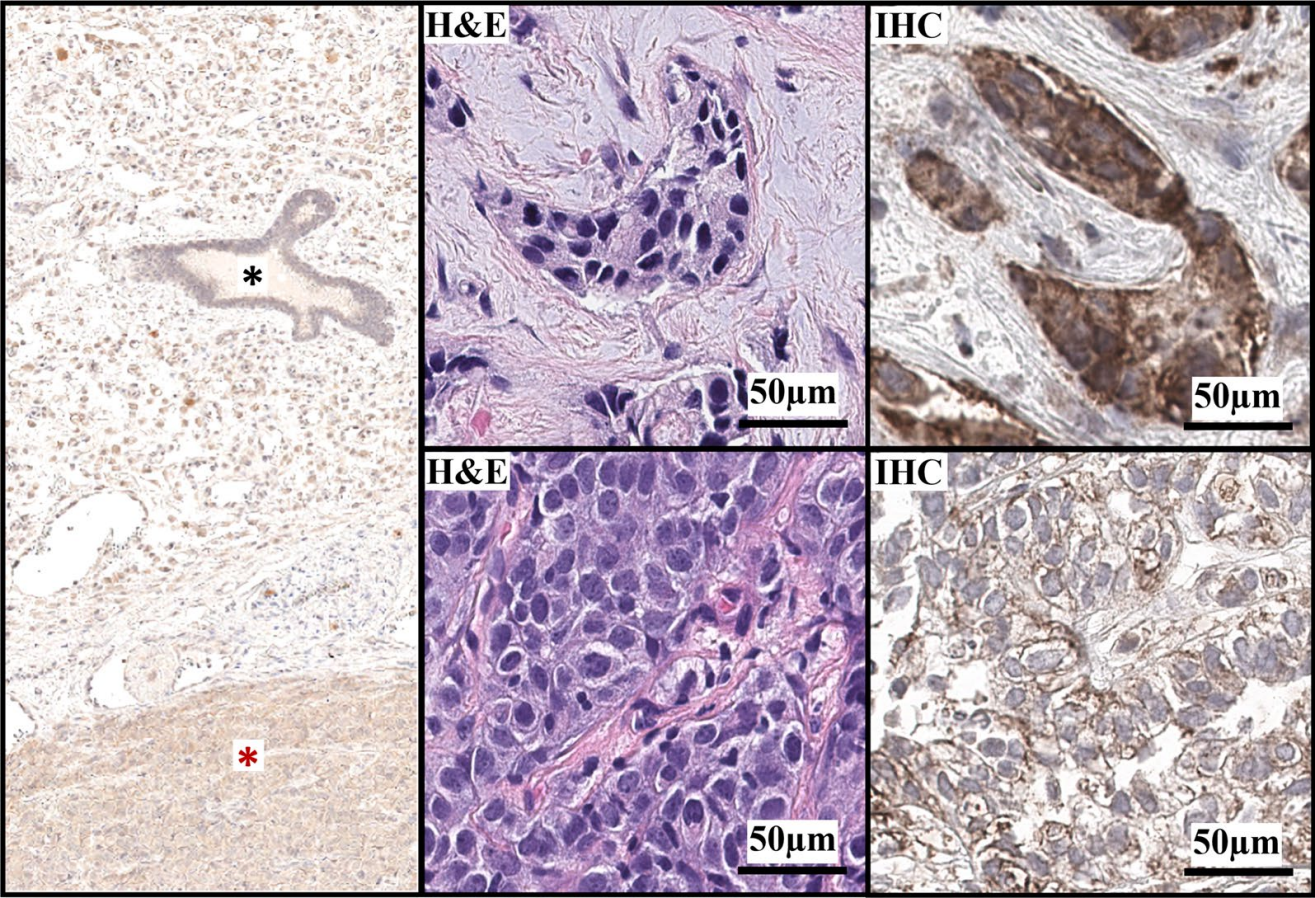
Representative baseline ROR1 immunohistochemistry. Representative ROR1 IHC of breast tissue obtained from patients BROR-01 and BROR-04. Left panel: Negative ROR1 staining in non-neoplastic mammary gland (black star) and in tumor area (red star). Top right panel: ROR1 high expressor with 99% of the cells highly positive (3+ = 5%; 2+ = 67%; 1+ = 27%; 0+ = 1%). Bottom right panel: ROR1 low expressor with 48% of the cells weakly positive (3+ = 0%; 2+ = 0.2%; 1+ = 48%; 0+ = 52%). *H&E* Hematoxylin and eosin, *IHC* immunohistochemistry

Eight patients had baseline formalin-fixed, paraffin-embedded tumor, fresh core biopsy samples, or recovery of cells from malignant ascites or pleural effusion available prior to treatment for assessment of ROR1 receptor protein expression by immunohistochemistry (Fig. 5). Five of 8 patients were ER^+^ or PR^+^/HER2^−^ at study entry, and 3/8 were ER^−^PR^−^/HER2^−^ at study entry. All eight baseline samples were found to express some level of ROR1. No correlation was found between baseline ROR1 expression and tumor response or tumor subtype (data not shown).

## Discussion

In this phase 1b study of heavily pre-treated patients with locally advanced, unresectable, or metastatic HER2^−^ breast cancer, the combination of zilovertamab and paclitaxel was found to be safe, well tolerated, and associated with partial response in 6/16 (38%) or stable disease in 6/16 (38%) patients. The most common AE attributed to zilovertamab was nausea. No DLTs, dose reductions, or discontinuations due to zilovertamab toxicity were observed. The safety profile of combination therapy reflected AEs most commonly attributed to paclitaxel. Strengths of our study include enrollment of heavily pre-treated patients. Limitations of our study include the small number of enrolled patients and lack of randomized comparison to standard of care paclitaxel.

Partial response was observed in 6/16 heavily pre-treated patients (38%), which is comparable to response to first-line therapy with paclitaxel in recent studies of patients with metastatic HER2^−^ breast cancer [17, 18]. In the later-line setting, one study of patients who had received up to two prior lines of chemotherapy for metastatic breast cancer demonstrated an overall response rate of 21.5% with weekly paclitaxel 80 mg/m^2^, suggesting that a response rate of 38% with combination therapy of zilovertamab and paclitaxel warrants further studies [19]. One patient, BROR-02, had an exceptional partial response lasting 48 weeks, with duration of zilovertamab and paclitaxel therapy for 23 weeks, followed by zilovertamab alone for 32 weeks. (Paclitaxel was discontinued due to paclitaxel-induced neuropathy.) Prior to enrollment, this patient had ER^+^/HER2^−^ breast cancer treated with curative intent chemotherapy and endocrine therapy in 2003. She developed bone metastases in 2016, was treated with a CDK 4/6 inhibitor and aromatase inhibitor for 2 years, and then developed a mesenteric lymph node metastasis that stained by IHC as ER^−^PR^−^HER2^−^ prior to her enrollment on this study. Of note, this patient’s cell-free DNA (cfDNA) testing using the Guardant platform obtained once while on trial-directed therapy revealed mutations in Cyclin D2 (*CCND2)* (3% cfDNA), *PIK3CA* (0.5% cfDNA), and Tumor Protein 53 (*TP53)* (0.4% cfDNA). After progression on zilovertamab, she received capecitabine for 2.5 years, and she is now receiving fulvestrant with alpelisib, with disease control for at least 1.5 years. Unfortunately, the patient had no baseline or on-study tumor biopsy for ROR1 immunohistochemical assay.

Pharmacokinetic analysis revealed a zilovertamab half-life of at least 28 days that did not decline with repeated therapy, indicating that zilovertamab did not induce neutralizing anti-zilovertamab antibodies. We noted that patients with malignant ascites or pleural effusion had lower levels of zilovertamab in ascites and pleura fluid than in plasma, which we speculate may be due to antibody pooling in large extravascular compartments that required repeated drainage.

Exploratory analysis of ROR1 protein expression by IHC in 8 patients with pre-treatment biopsies or paraffin blocks revealed all tumor specimens expressed some level of ROR1. We did not observe an apparent correlation between baseline expression level of ROR1 and the magnitude of anti-tumor response. Study limitations included the lack of tissue at baseline and on study treatment, precluding assessment of ROR1 protein expression or gene expression over time. Additional limitations included lack of correlation of response with baseline ROR1 level, lack of ROR1 gene expression assayed in tumor specimens, and lack of consensus of which antibodies are best suited for detection of ROR1 on formalin-fixed cancer tissue. Analysis of ROR1 protein expression by immunohistochemistry is limited by the specificity and availability of mAbs that reliably detect this protein in fixed-tissue specimens and is the subject of ongoing research.

Prior studies from our group demonstrated an association of ROR1-signaling with stem cell features, epithelial–mesenchymal transition, tumor proliferation, and metastases in preclinical models [13]. Preclinical and phase 1 studies of zilovertamab in CLL demonstrated tolerability and anti-tumor activity with inhibition of ROR1-signaling, cancer-stemness gene expression, and expression of genes induced by activation of extracellular signal-regulated kinase 1/2 (*ERK1/2*), nuclear factor-kappa B (*NF-κB*), and nuclear factor erythroid 2-related factor 2 (*NRF2*) [13]. Preclinical breast cancer studies demonstrated that zilovertamab repressed expression of genes associated with breast cancer stemness, impaired metastasis, and inhibited re-engraftment in immunodeficient mice [14]. Additionally, zilovertamab in combination with paclitaxel had at least additive anti-tumor effects, further justifying this clinical study [14].

Our group has evaluated the pre-treatment transcriptome database of 989 high risk early breast cancer patients treated on the I-SPY2 platform with novel agents and neoadjuvant chemotherapy. Among breast cancer patients with higher residual disease burden after neoadjuvant therapy for HR^+^/HER2^−^ breast cancer, patients with high-level expression of *ROR1* had significantly worse event-free survival than those with low-level expression of *ROR1* [12]. These results suggest that further studies of high-level expression of *ROR1* are justified to determine if it may identify patients appropriate for investigational studies of ROR1-targeted agents.

Zilovertamab is under evaluation in CLL and mantle cell lymphoma in combination with ibrutinib (NCT03088878). Our group has developed a ROR1 antibody conjugated to MMAE that was effective in a Richter’s syndrome mouse model [20]. This compound, zilovertamab vedotin (previously VLS-101), was found to have clinical activity and no unexpected toxicities in heavily pre-treated patients with lymphoid cancer [21]. Zilovertamab vedotin is under study in hematologic malignancies (NCT03833180) and in solid tumors (NCT04504916).

In summary, the combination of zilovertamab and paclitaxel was safe and well tolerated in heavily pre-treated advanced breast cancer. Further evaluation of ROR1 expression and ROR1 targeting in breast cancer is warranted.

## Conclusions

ROR1 is an onco-embryonic antigen expressed on neoplastic cells of a variety of different cancers, including breast cancer, but not on most normal postnatal tissues, making it a potential target for anti-cancer therapy. ROR1-signaling is associated with epithelial–mesenchymal transition, tumor proliferation, and metastases. Inhibition of ROR1-signaling enhances the anti-tumor activity of paclitaxel in preclinical models. Clinical studies have demonstrated that the humanized anti-ROR1 mAb zilovertamab is safe and effective in inhibiting ROR1-signaling in patients with ROR1-positive leukemia. Sixteen patients with advanced HER2^−^ breast cancer enrolled in a phase 1b trial to study zilovertamab in combination with paclitaxel. Pharmacokinetic studies revealed zilovertamab had a plasma half-life of at least 28 days. Treatment with the combination of zilovertamab and paclitaxel was well tolerated and effective in inducing a partial response in 6/16 (38%) or stable disease in 6/16 (38%) patients. These results justify further clinical studies targeting ROR1 for treatment of patients with advanced breast cancer.

## Data Availability

The data generated in this study are available upon reasonable request from the corresponding author.

## Abbreviations

AE: Adverse event
cfDNA: Cell-free DNA
CLL: Chronic lymphocytic leukemia
CT: Computed tomography
CTCAE: Common Terminology Criteria for Adverse Events
DLT: Dose limiting toxicity
ECOG: Eastern Cooperative Group
HRPP: Human Research Protections Program
IHC: Immunohistochemistry
mAb: Monoclonal antibody
PDX: Patient-derived xenograft
PET/CT: Positron emission tomography/computed tomography
PFS: Progression-free survival
PR: Partial response
RECIST: Response Evaluation Criteria for Solid Tumors
ROR1: Receptor tyrosine kinase-like orphan receptor 1
TRAE: Treatment-related adverse event
