# Correction: a phase 1b study of zilovertamab in combination with paclitaxel for locally advanced/unresectable or metastatic HER2-negative breast cancer

**DOI:** 10.1186/s13058-024-01805-w

**Published:** 2024-03-13

**Authors:** Rebecca A. Shatsky, Hemali Batra-Sharma, Teresa Helsten, Richard B. Schwab, Emily I. Pittman, Minya Pu, Elizabeth Weihe, Emanuela M. Ghia, Laura Z. Rassenti, Alfredo Molinolo, Betty Cabrera, James B. Breitmeyer, George F. Widhopf II, Karen Messer, Catriona Jamieson, Thomas J. Kipps, Barbara A. Parker

**Affiliations:** 1 Moores Cancer Center, University of California San Diego, 3855 Health Sciences Drive Mail Code 0987, La Jolla, San Diego, CA 92093 USA; 2 Department of Medicine, University of California San Diego, La Jolla, San Diego, CA USA; 3 Herbert Wertheim School of Public Health, University of California San Diego, La Jolla, San Diego, CA USA; 4 Department of Radiology, University of California San Diego, La Jolla, San Diego, CA USA; 5 Center for Novel Therapeutics, University of California San Diego, La Jolla, San Diego, CA USA; 6 University of California San Diego California Institute for Regenerative Medicine Alpha Clinic, La Jolla, San Diego, CA USA; 7 Oncternal Therapeutics, Inc., San Diego, CA USA; 8 Sanford Stem Cell Institute, University of California San Diego, La Jolla, San Diego, CA USA

**Correction to: Shatsky et al. Breast Cancer Research (2024) 26:32** 10.1186/s13058-024-01782-0

The original article contained three typesetting errors. “Her2-negative” in the title should have been written as “HER2-negative”. Hence, the title should read: A phase 1b study of zilovertamab in combination with paclitaxel for locally advanced/unresectable or metastatic HER2-negative breast cancer, as shown in this Correction Article. 

In the Background section of the original article, on page 2, paragraph 2, there was a misspelling of “immuneodeficient”. The correct spelling is “immunodeficient.”

Also on page 2, in the section on Patient Selection, there was a misspelling of “HERer2”. The correct spelling is “HER2^-^” as displayed throughout the manuscript.

The original article [1] has been updated.
